# Distributed Hybrid Two-Stage Multi-Sensor Fusion for Cooperative Modulation Classification in Large-Scale Wireless Sensor Networks

**DOI:** 10.3390/s19194339

**Published:** 2019-10-08

**Authors:** Goran B. Markovic, Vlada S. Sokolovic, Miroslav L. Dukic

**Affiliations:** 1 School of Electrical Engineering, University of Belgrade, Bul. kralja Aleksandra 73, 11120 Belgrade, Serbia; dukic@etf.bg.ac.rs; 2 University of Defence in Belgrade, Military Academy, Generala Pavla Jurišića Šturma 33, 11000 Belgrade, Serbia; vlada.sokolovic@va.mod.gov.rs

**Keywords:** cognitive radio networks, data fusion, feature fusion, hybrid fusion, multi-sensor fusion, modulation classification, wireless sensor networks

## Abstract

Recent studies showed that the performance of the modulation classification (MC) is considerably improved by using multiple sensors deployed in a cooperative manner. Such cooperative MC solutions are based on the centralized fusion of independent features or decisions made at sensors. Essentially, the cooperative MC employs multiple uncorrelated observations of the unknown signal to gather more complete information, compared to the single sensor reception, which is used in the fusion process to refine the MC decision. However, the non-cooperative nature of MC inherently induces large loss in cooperative MC performance due to the unreliable measure of quality for the MC results obtained at individual sensors (which causes the partial information loss while performing centralized fusion). In this paper, the distributed two-stage fusion concept for the cooperative MC using multiple sensors is proposed. It is shown that the proposed distributed fusion, which combines feature (cumulant) fusion and decision fusion, facilitate preservation of information during the fusion process and thus considerably improve the MC performance. The clustered architecture is employed, with the influence of mismatched references restricted to the intra-cluster data fusion in the first stage. The adopted distributed concept represents a flexible and scalable solution that is suitable for implementation of large-scale networks.

## 1. Introduction

The recent advances of wireless networks, which consist of spatially distributed transceivers or sensor nodes, e.g., wireless sensor network (WSN), cognitive radio networks (CRN), and general ad hoc wireless networks, endorse successful application of cooperative paradigms in different communication and processing applications. Some examples of such applications are cooperative localization [1,2], cooperative spectrum sensing [3,4,5], and cooperative communication [6,7,8]. The cooperativeness fundamentally exploits the redundancy in the sensing process or the radio signal transmission/reception by using spatially distributed sensors to facilitate reliability and performance improvements in the signal transmission, detection, localization, classification, sensing, or processing in general. The proper use of cooperative solutions relies on the successful communication and collaboration between the engaged sensor and radio nodes, which can be implemented via centralized or distributed approaches. Centralized approaches are generally considered to achieve a better performance than distributed ones, but at the cost of increased energy consumption, communication requirements (higher traffic load, more complex medium access, and routing, etc.) and computational complexity. In the case of large-scale distributed wireless networks, which are usually characterized with complex multi-hop communication, the communication burden, and the high energy consumption related to the centralized approach severely limits the efficiency of centralized solutions. This is especially true for WSN consisting of simple sensor nodes with low energy reserves. Therefore, the distributed approach presents a more viable solution for large-scale ad hoc wireless networks.

The modulation classification (MC) of a received radio signal emitted by a non-cooperative and completely unknown radio transmitter represents a difficult problem. The non-cooperative nature of the MC process results in a highly restricted knowledge about the received signal. Therefore, the classifier must rely only on the short signal sample and the blind signal processing. The MC problem becomes even more difficult in the case of unknown signal reception with low signal-to-noise ratio (SNR) or over frequency-selective fading channels. Hitherto, many different MC solutions were proposed based on using a single receiver, e.g., [9,10,11,12,13,14,15,16,17,18]. Among them, there are various likelihood based (LB) MC solutions that achieve near-optimal performance under the assumed ideal conditions [9,10,11], but also exhibit a decline in MC performance due to the signal parameter estimation errors. Different complex LB classifiers are proposed for the signal reception over the flat fading channels, while even more complex solutions are needed in the case of frequency-selective fading channels in order to combat the multipath fading (MPF) channel influence [9,10,11]. In contrast, the less complex feature based (FB) MC solutions offer suboptimal MC performance compared to the LB classifiers. Among these, the cumulant-based classifiers gained much attention [14,15,16,17,18], due to their considerably lower complexity, high robustness on parameter estimation errors, and the inherent suppression of the MPF channel influence through the blind channel estimation [14,15,16,17]. However, the performance of these MC solutions, which rely on signal reception with single sensor, significantly decrease due to the variable quality of signal reception, especially in the case of low reception level and the influence of dispersive MPF radio propagation environments.

Although modulation classification has a number of traditional applications, e.g., radio spectrum surveillance and management, the more important new ones emerge by the advance of adaptive modulation, software defined radio, and CRN concepts [3]. In addition, these new communication concepts and technologies, e.g., CRN and WSN, provide a network environment with scattered sensors that facilitate a development of cooperative MC schemes. Trends in spectrum surveillance and electronic warfare systems suggest the use of densely distributed sensors instead of classical monitoring stations. WSN and ad hoc wireless networks appear as the obvious networking solutions, consisting of sensor nodes distributed in the given area, embedded in moving vehicles or in unmanned aerial vehicles. These networks could be deployed for different applications that demand detection, identification, MC, and localization of radio signals of unknown transmitters. In these specific applications of WSN technology, sensor nodes should be equipped with an additional receiver (i.e., radio sensor) that monitors specific frequency bands, besides the usual communication interface responsible for wireless communication. Sensor nodes in WSN are generally constrained in power supply, processing power, and memory. Thus, specific WSN solution designed for cooperative sensing, detection, MC, and localization must also support energy efficiency and low complexity processing, and require minimum communication in order to perform given tasks. In CRN, cooperative MC can be used as an integral part of the transmitter identification process and deployed after spectrum sensing. In addition, the self-configured software defined CRN are envisioned that employ MC for link establishment purposes. As MC has secondary importance in CRN, the low power consumption, processing complexity, and communication demands are needed.

The results of recent studies [3,19,20,21,22,23,24,25,26,27,28,29,30,31,32,33,34,35,36,37,38] indicate that significant MC performance gains are achievable by using multiple sensors (instead of a single sensor reception) in a cooperative manner, even for the dispersive MPF environments. In essence, cooperative MC solutions employ multiple uncorrelated observations of the unknown signal in order to gather more complete information about it, compared to the single sensor reception, and later use this additional information to refine the MC decision through the fusion process.

A distributed processing for cumulant estimation in CRN/WSN is observed in [19], in order to improve the cumulant estimation quality compared to single sensor estimation, but has a high communication demand and becomes unstable in dispersive MPF channels. The cooperative centralized hard decision fusion (HDF) is considered in [20,21,22,23]. The joint cooperative MC and spectrum sensing solution is proposed in [20], with a high complexity iterative decision process using cyclic spectrum analysis. Due to the high communication demands it is not suitable for large-scale networks. The optimal HDF based on local decisions made in sensors by using the LB classifier [21,23], or the cumulant-based classifier [22,23] can be found in literature. The proposed optimal HDF is realized through the combination of local decisions in the fusion centre (FC) based on the *a priori* known probabilities of correct/incorrect decisions used as references. However, these classifiers strongly depend on the reference mismatch [24,25,26]. The several centralized data fusion (DaF) and soft decision fusion (SDF) cooperative classifiers [24,25,26] were also proposed, in which the final decision is made by combining local cumulant estimates from sensors, based on the *a priori* knowledge of cumulant estimate means and variances as references. These cumulant based solutions are shown to be more robust to the reference mismatch. Moreover, a simple centralized DaF scheme is proposed in [27], with the final decision made based on the maximum ratio combining or equal gain combining of the local decision statistics calculated by LB classifier in each sensor. Finally, simple centralized hybrid DaF/HDF cumulant-based solutions were proposed in [28,29]. Moreover, an interesting cumulant-based cooperative MC solution for flat fading channels is proposed in [36]. The hierarchical cumulant-based cooperative MC scheme with feature-level cooperative classification framework using maximum likelihood combining algorithm is proposed in [37,38].

The centralized signal fusion cooperative MC solutions are considered [23,30,31,32,33,34,35], in which the complete received symbol sequences are transmitted to the FC. The complex signal fusion process is applied before the cumulant-based MC [23,30], or the signal fusion is performed as a high complexity iterative optimization process [31,32,33,34,35], based on expectation maximization [31,32,33,34], or some other suboptimal and less complex processing [35], on the received symbol sequences from all sensors. The likelihood-based cooperative signal fusion solutions [23,31,32,33,34,35], are shown to achieve a nearly optimal performance and thus outperform the centralized cooperative cumulant-based solutions. However, these centralized signal fusion solutions, require that a same symbol sequence is received and separately delivered to the FC by all sensors [23,31,32,33,34,35]. This results in significant communication load between the sensors and the FC, and demand a certain level of sensing synchronization on the network level (which adds further complexity). Furthermore, since modulation classification is not the main function of large-scale CRN, and constrained nodes are used in WSN, the high communication demands and complexity significantly limits applicability of the centralized signal fusion solutions in these networks. Therefore, these solutions can be successfully applied if the sensing network is in the vicinity of the FC (or multiple antennas connected to the FC are used), and do not present a viable choice for large-scale ad hoc networks such as WSN and CRN.

Furthermore, all cooperative MC solutions with centralized fusion depend on the certain reference values used in the fusion process as the measure of the local MC results reliability, e.g., confusion matrices in HDF [21,22,23], the cumulant estimate means and/or variances in DaF [24,25,26], or the assumed likelihoods expressions in the LB approach [22,27]. The non-cooperative nature of the MC process inherently induces a large MC performance loss for the centralized fusion-based solutions due to the unreliable measure of quality (references) for the obtained MC results in individual sensors. In fact, the cause of this performance loss is the partial loss of information due to the unreliable references while performing the centralized fusion [24,25,26].

In this paper, we propose a novel distributed two-stage fusion concept for the cooperative MC using multiple sensors. The proposed cooperative MC solution consists of a first stage, in which neighbouring sensors form clusters and use data fusion to make decisions on the cluster level, while in the second stage these cluster decisions are gathered and used to make a final decision. We here show that the proposed two-stage fusion facilitates preservation of information during the fusion process and thus considerably improves the performance of such cooperative MC scheme. The performance improvement is realized through mitigation of reference mismatch that causes the information loss, by restricting its influence to intra-cluster fusion at the first stage. In most of the previous work in the area, except [21,24,25,26,28], multi-sensor fusion is performed under the assumption that all sensors receive the signal over similar frequency-selective MPF channels (i.e., with the same channel impulse response length) and with similar SNR. Therefore, in order to model practical application conditions, we have defined three different sensor network scenarios depending on the sensors spacing around the transmitter, in which each sensor receives the unknown signal over uncorrelated MPF channels with different SNR (from zero dB to 20 dB) and MPF channel features (i.e., the different channel impulse response length). In addition, it should be noted that here the proposed distributed fusion possesses a potential to mitigate the communication complexity and energy consumption issues that impose significant limitations to centralized fusion, especially in the case of large-scale networks such as WSN and CRN. This problem, although important is seldom analysed in literature, i.e., in [38]. In order to implement here the proposed distributed cooperative MC, a detailed specification of clustering protocol, data exchange, and distributed computation protocol is needed. However, in this paper we are focused on the theoretical framework rather than specific protocol details. The aim of this work is to properly introduce a distributed cooperative MC concept and demonstrate its classification performance. Therefore, a detailed network framework and protocol proposal is beyond the scope of this paper.

This paper is organized as follows. Section 2 presents a short review of the cumulant-based centralized fusion for cooperative MC in frequency-selective MPF channels, with the focus on cumulant estimate quality under different conditions (i.e., SNR value, MPF channel features, signal sample length). In Section 3, we present the basic working principles and ideas of the proposed distributed two-stage hybrid fusion for cooperative MC solution, with the strict definition of the proposed scheme. Section 4 describes the settings, application scenarios, and results of the numerical analysis carried out through comprehensive Monte Carlo simulations in order to estimate MC performance of the proposed and reference solutions. This section gives an overview of the most interesting results for the all considered sensor network scenarios, as well as the appropriate discussion. Finally, the conclusions and remarks are presented in Section 5.

## 2. Centralized Fusion for Multi-Sensor Cooperative MC

**Figure 1:**
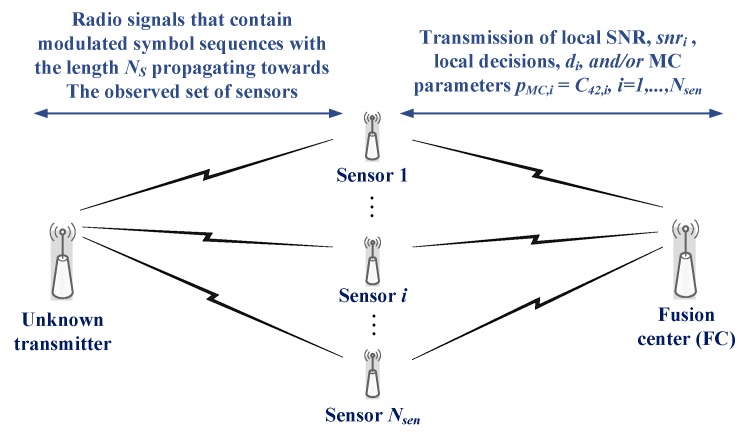
The general scheme of cooperative modulation classification (MC) with centralized fusion [25].

Here, we observe the centralized fusion for cooperative modulation classification MC as proposed and studied in previous work in the area [20,21,22,23,24,25,26,27,28,29,36,37,38], with the general scheme given in Figure 1. The $N_{sen}$ sensors independently receive the modulated signal, which is emitted by the same unknown transmitter by using an unidentified modulation type belonging to the known set, $m\in M_{mod}=\left\{m_{1},\cdots ,m_{M}\right\}$, over the uncorrelated dispersive multipath fading (MPF) channels. It is assumed that the sensor specific local signal-to-noise ratio (SNR) values, $snr_{i},i=1,\cdots ,N_{sen}$, are known (i.e., reliably estimated) and that each sensor collects the sample sequence of $N_{S}$ modulated symbols, $y_{i}(n),\;n=1,\cdots ,N_{S}$. The same cumulant-based MC process is applied on the sample sequence at each sensor, as described in Section 2.2, to produce a local cumulant estimate, $C_{42,i}$, or a local hard decision, $d_{i},\;i=1,\cdots ,N_{sen}$. The local MC results, i.e., the local cumulant estimates, $C_{42,i}$, or local decisions, $d_{i}$, and the corresponding local SNRs, ${\mathrm{snr}}_{i}$, are collected at the fusion centre (FC), where the final decision regarding the modulation type is made. Error-free communication between the FC and sensors is assumed, which corresponds to the best cooperative MC performance. The performance deterioration due to the specific communication and wireless network scenarios can be evaluated, but it is beyond the scope of this study. It should be noticed that here we use the term sensor as a general term for the network node of cognitive radio network (CRN), wireless sensor network (WSN), or general distributed network equipped with the receiver (radio sensor) responsible for the reception (sensing) of the unknown signal, and which can possess separate communication interface (e.g., in WSN) for wireless communication and exchange of data with other nodes in the network.

### 2.1. Cumulant-Based MC in Dispersive MPF Channels

For the unknown signal reception in the dispersive MPF channel, the received baseband symbol sample sequence at the i-th sensor, $y_{i}\left(n\right),\;i=1,\cdots ,N_{sen}$, can be defined [14,15,16,17,18,24,25,26], as

(1) $$y_{i}\left(n\right)=\sum_{k=0}^{L_{i}-1}h_{i}\left(k\right)x_{i}\left(n-k\right)+g_{i}\left(n\right),$$

where $h_{i}\left(k\right),k=0,\cdots ,L_{i}-1$, are the MPF coefficients of an unknown dispersive MPF channel of length *L_i_*, the $x_{i}\left(n\right)$ is the *n*-th received symbol of the observed signal, while the $g_{i}\left(n\right)$ is the *n*-th sample of complex additive white Gaussian noise (AWGN) process with a zero mean and a variance $\sigma_{g,i}^{2}$. The corresponding SNR is defined as $E\{x_{i}^{2}(n)\}/\sigma_{g,i}^{2}$ [14,15,16,17].

The local MC results at the observed i-th sensor, $i=1,\cdots ,N_{sen}$, are produced based on the normalized fourth-order cumulant of the emitted symbol sequence, $C_{42}$ [14], i.e., using the local cumulant estimate $C_{42,i}$, calculated with the correction factor $\beta_{i}$ that represents the impact of the unknown MPF channel,

(2) $$C_{42,i}=\frac{N_{S}\sum_{k=0}^{N_{s}-1}{\left|y_{i}\left(k\right)\right|}^{4}-{\left[\sum_{k=0}^{N_{s}-1}y_{i}^{2}\left(k\right)\right]}^{2}-2{\left(\sum_{k=0}^{N_{s}-1}{\left|y_{i}\left(k\right)\right|}^{2}\right)}^{2}}{\beta_{i}\times {\left[\sum_{k=0}^{N_{S}-1}{\left|y_{i}\left(k\right)\right|}^{2}-N_{S}\sigma_{g,i}^{2}\right]}^{2}},$$

(3) $$\beta_{i}=\frac{\sum_{k=0}^{L_{i}-1}{\left|h_{i}\left(k\right)\right|}^{4}}{{\left[\sum_{k=0}^{L_{i}-1}h_{i}^{2}\left(k\right)\right]}^{2}}.$$

The non-cooperativeness of the MC process inherently prevents *a priori* knowledge of the actual MPF coefficients. Therefore, the $\beta_{i}$ and the local cumulant estimate $C_{42,i}$ are estimated from the locally received baseband sequence, $y_{i}\left(n\right)$, $n=1,\cdots ,N_{S}$, of length $N_{S}$, with the MPF coefficients estimated using the blind channel estimation methods (BCEM) proposed in [16,17], which are found to be suitable for dispersive MPF channels with a dominant path [16,25,26]. Since the method proposed in [17] has shown a slightly better behaviour, in this study we used this method (marked as BCEM) for the local cumulant estimation. Moreover, here we observe the ideal channel estimation model (ICEM), with the local MPF coefficients and channel length $L_{i}$ assumed *a priori* known, in order to model/observe the ideal channel equalization, i.e., the best case of local cumulant estimation.

In the case when the local decisions are needed, after the local cumulant estimate is obtained a hard decision rule defined in [14] is applied, with the decision thresholds set as arithmetic means of neighbouring reference cumulant means for the possible modulation types. In Table 1, the theoretic means of the fourth-order cumulant for some phase shift keying (PSK) and quadrature amplitude modulation (QAM) signals are given [14]. It is shown in [24,25,26] that these theoretic values are optimal for signal reception in AWGN channels or when the ICEM is used. However, when BCEM is applied for the most dispersive MPF channels with a dominant path, the correction factor $\beta_{i}$ in equation (3) is usually (in average) underestimated [24,25,26]. Consequently, the actual cumulant means are in fact stirred toward the larger absolute values.

We here consider a typical model for dispersive MPF channel with a dominant path that is usually observed in similar studies [14,15,16,24,25,26]. Thus, the channel length $L_{i}$ and the MPF coefficients, $h_{i}\left(k\right),k=0,\cdots ,L_{i}-1$, for each sensor represent the independent zero-mean Gaussian random variables with variance $\sigma_{h,i}^{2}=0.05$, and with $h_{i}\left(0\right)=1$ [14,15,16,24,25,26]. This MPF channel model corresponds to the line-of-sight reception over dispersive MPF channels, as well as for the reception with the non-ideal channel equalization and the residual channel impact modelled with the coefficients $h_{i}\left(k\right),k>0$ [14].

### 2.2. The Fusion Methods and the Joint Cumulant Estimate Correction

The fusion process can be realized by using the local hard decisions, $d_{i}$, thus defining hard decision fusion (HDF) methods, by using the local cumulant estimates $C_{42,i}$, thus defining data fusion (DaF) methods, or we can combine these two concepts in order to define soft decision fusion (SDF) methods. Due to the non-cooperative nature of the MC process, the channel state information must be assumed unknown. Hence, the local SNR, marked as $snr_{i}$, represents the only available quality measure for the locally received signal at the *i*-th sensor, $i=1,\cdots ,N_{sen}$, and henceforth the local cumulant estimate quality and local decisions reliability as well. In this section, we only give a brief summary of observed fusion methods for the centralized fusion for cooperative MC [22,24,25,26]. These fusion methods, proposed in previous studies [22,24,25,26], are chosen due to their good behaviour in the context of centralized fusion for cooperative MC solutions [22,24,25,26].

The optimal HDF method (OHDF) is proposed in [22], with the decision rule derived to make a final decision $M_{F,\mathrm{OHDF}}$, as the most likely cause for the local decisions, $d_{i}$, $i=1,\cdots ,N_{sen}$, made under the observed local SNR values, $snr_{i},\;i=1,\cdots ,N_{sen}$, and defined as

(4) $$M_{F,OHDF}=\underset{m_{n}\in M_{mod}}{\underbrace{argmax}}\langle \prod_{i=1}^{N_{sen}}\frac{p\left(d_{i}|\left(m_{n},snr_{i}\right)\right)}{\sum_{k=1}^{M}p\left(d_{i}|\left(m_{k},snr_{i}\right)\right)}\rangle ,$$

where $p\left(d_{i}|\left(m_{n},snr_{i}\right)\right)$ is a probability of local decision $d_{i}$ made for a given local SNR, $snr_{i}$, when the real modulation type is $m=m_{n}$. In order to implement OHDF the *a priori* knowledge of the reference confusion matrices (CM), i.e., the set of probabilities $p\left(m_{i}|\left(m_{n},snr_{i}\right)\right),\;\forall \left(m_{i},m_{n}\right)\in M_{mod}$, is needed for all local SNR values. Moreover, the reference cumulant means are needed in order to set the local decision thresholds at each sensor [14,24,25,26].

The DaF methods [24,25,26] are derived and shown to be effective under the assumption of high quality local cumulant estimates, $C_{42,i}$, $i=1,\cdots ,N_{sen}$, i.e., when the MPF channel influence is adequately suppressed with the correctly estimated $\beta_{i}$ in Equation (2). In that case, the cumulant estimate at the i-th sensor, $C_{42,i}$, can be approximately modeled as a normally distributed random variable, with a probability density function (PDF) $N\left(C_{42}^{m}\left(snr_{i}\right),\sigma_{m}^{2}\left(snr_{i}\right)\right)$, [24,25,26] where $C_{42}^{m}\left(snr_{i}\right)$ is the actual mean and the $\sigma_{m}^{2}\left(snr_{i}\right)$ is the actual variance for a given $snr_{i}$, MPF channel probability density function and the real modulation type m. As we observe the independent local signal reception through the uncorrelated MPF channels, the local cumulant estimates, $C_{42,i}$, represent mutually independent random variables. By applying the logarithmic likelihood ratio test, the joint decision fusion (JDF) method is derived in [24], with a decision rule given as

(5) $$M_{F,JDF}=\underset{m_{n}\in M_{mod}}{\underbrace{argmax}}\langle \sum_{i=1}^{N_{\mathrm{sen}}}\ln\left(\frac{1}{\sigma_{m_{n}}\left(snr_{i}\right)}\right)-{\frac{\left(C_{42,i}-C_{42}^{m_{n}}\left(snr_{i}\right)\right)}{2\sigma_{m_{n}}^{2}\left(snr_{i}\right)}}^{2}\rangle .$$

In order to implement the JDF method, the reference cumulant means and reference cumulant variances should be *a priori* known for the all possible modulation types ($m_{n}\in M_{mod}$), local SNR values, and the specific MPF channels states (defined with the channel length $L_{i}$ and the MPF coefficients PDF). Therefore, we can apply either the theoretic cumulant means, as in Table 1, or the estimated actual cumulant means that are in accordance with the given SNR and MPF channel conditions, while the reference cumulant variances must be properly estimated under the same conditions in accordance with the selected reference cumulant means. However, the good performance of the JDF method is expected only when the initial assumption of the high quality local cumulant estimate is satisfied.

The soft decision vector decision fusion (SDVDF) method is proposed in [26], which introduces additional weighting of the local decisions with the soft decision vector, $s_{i}=\left[s_{i,1},\cdots ,s_{i,M}\right]$, as the measure of the conditional probabilities that cumulant estimate $C_{42,i}$ is acquired when the actual modulation type is $m_{n},\;n=1,\cdots ,M,$ under the local SNR values $snr_{i}$. The SDVDF method decision rule is given as [26]

(6) $$M_{F,SDVDF}=\underset{m_{n}\in M_{mod}}{\underbrace{argmax}}\langle \prod_{i=1}^{N_{sen}}\sum_{j=1}^{M}s_{i,j}\times \frac{p\left(m_{j}|\left(m_{n},snr_{i}\right)\right)}{\sum_{k=1}^{M}p\left(m_{j}|\left(m_{k},snr_{i}\right)\right)}\rangle ,$$

(7) $$s_{i,j}=\frac{p\left(C_{42,i}|\left(m_{j},snr_{i}\right)\right)}{\sum_{k=1}^{M}p\left(C_{42,i}|\left(m_{k},snr_{i}\right)\right)},$$

(8) $$p\left(C_{42,i}|\left(m_{n},snr_{i}\right)\right)=\frac{1}{\sqrt{2\pi }\sigma_{m}\left(snr_{i}\right)}\times \;\exp\left(-{\left(\frac{C_{42,i}-C_{42}^{m_{n}}\left(snr_{i}\right)}{\sqrt{2}\sigma_{m}\left(snr_{i}\right)}\right)}^{2}\right),\;i=1,\cdots ,N_{sen}.$$

Obviously, the implementation of the SDVDF method requires the knowledge of the appropriate reference CMs, reference cumulant means, and reference cumulant variances.

In the JDF and SDVDF methods, the FC has at its disposal multiple uncorrelated local cumulant estimates, which in fact represent independent realizations of the cumulant estimation process, i.e., estimation of $C_{42}$ corresponding to the emitted sequence, under different local MPF channel and SNR conditions. Based on this, the joint cumulant estimate correction (JCEC) is proposed in [25], with the joint cumulant estimation using local SNRs as a measure of the local cumulant estimate quality, and the correction of all local cumulant estimates based on this joint estimate and reference cumulant means. It is shown [25,26] that the modified JDF and SDVDF methods applied after the JCEC by using these corrected local cumulant estimates, marked here, respectively as JDF+JCEC and SDVDF+JCEC, achieve considerably better MC performance than the original JDF and SDVDF methods in the case of low cumulant estimate quality, i.e., for lower SNR values, short sample sequences (small $N_{S}$), and highly dispersive MPF channels (i.e., bigger MPF channel lengths).

### 2.3. The Actual Cumulant Estimate Quality and References Estimation

The fusion process for all the considered fusion methods described in Section 2.2 demand the knowledge of certain references, i.e., the reference cumulant means and variances and/or reference CMs that correspond to the actual MPF channel parameters (L, SNR, channel PDF type, and parameters) and processing parameters ($N_{S}$, channel estimation method, i.e., ICEM or BCEM) and a given modulation set $M_{mod}$. We performed an extensive Monte Carlo based iterative estimation process in MatLab environment in order to determine these references for the cumulant-based MC with ICEM or with BCEM used (separately) and the MPF channel model defined in Section 2.1. These references are adequately estimated for the mixture of PSK and QAM modulated signals from the set $M_{mod}=\left\{BPSK,\;QPSK,\;16QAM,\;64QAM\right\}$ that are generated as the normalized unit energy zero-mean random processes with the randomly generated symbol sequences x(n), $n=1,\cdots ,N_{S}$, where $N_{S}\in \left\{500,\;1000,\;2000,\;4000\right\}$, and for the SNR∈[−5 dB,20 dB]. In order to examine the effect of different time-dispersion level introduced by the MPF channel, the MPF channels with the channel lengths L∈{2,⋯, 10} were observed, with the channel coefficients randomly generated according to the defined channel model. The actual cumulant (estimate) means are obtained for all the possible parameter combinations by averaging over independent trials (5000 trials by iteration until the convergence of the estimated values is achieved in the subsequent iterations). The actual cumulant (estimate) variances and the actual CMs are estimated separately for the estimated actual cumulant means and the theoretic cumulant means. In total, about 30,000,000 trials were executed.

**Figure 2:**
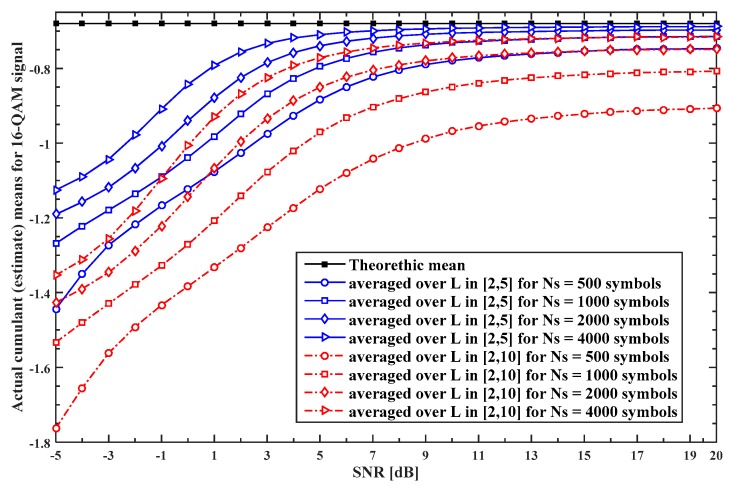
The actual cumulant (estimate) means for the 16QAM signal averaged over L∈{2,⋯, 5} and L∈{2,⋯, 10} as a function of signal-to-noise ratio (SNR) for the MC with the blind channel estimation method (BCEM).

**Figure 3:**
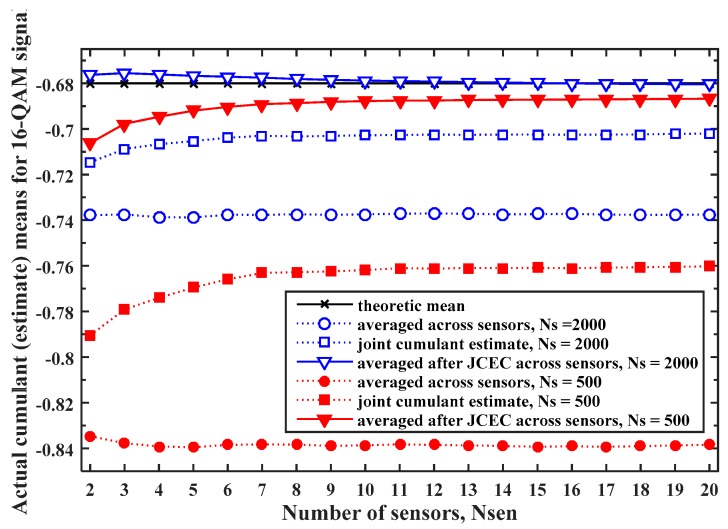
The actual cumulant (estimate) means for the 16QAM signal, with and without the joint cumulant estimate correction (JCEC) used for the MC with the blind channel estimation method (BCEM), averaged over different number of sensors with SNR=(10±2) dB and random L∈{2,⋯, 5}.

The similar behaviour of the cumulant estimate quality regarding the MPF channel and processing parameters is obtained for all the modulated signals in Table 1. Therefore, as an illustration, we here use some results for the 16QAM signal, presented in Figure 2 and Figure 3.

The actual cumulant means for the 16QAM signal averaged over L∈{2,⋯, 5} and L∈{2,⋯, 10} are shown on Figure 2 as a function of SNR for MC with BCEM used. As evident in Figure 2, the actual cumulant means obtained for the given MPF channel when L∈{2,⋯, 5} have a small deviation from theoretic values until SNR drops under 3–9 dB (depending on the sample length) and this deviation rises as SNR further decreases. However, if L∈{2,⋯, 10} a considerably larger shift from the theoretic mean value exists for all SNR values. Therefore, the cumulant estimate quality, and thus MC performance, is expected to improve with the rise of the sample sequence length $N_{S}$ and SNR, and to degrade with the rise of the MPF channel length L, i.e., with a higher level of dispersion in time introduced by the MPF channel. Moreover, in the case when the MC with ICEM is observed, i.e., for idealized scenario that is unattainable in practice, we found that the actual cumulant estimate for all signals does not depend on the MPF channel state and is almost equal to the theoretic mean for SNR larger than 5 dB, while for SNR smaller than 5 dB there is a slowly rising shift from the theoretic means as SNR further decreases.

In order to evaluate the cumulant estimate quality improvement when JCEC is used in FC for MC with BCEM, the actual cumulant (estimate) means averaged over different number of sensors with SNR=(10±2) dB and randomly generated L∈{2,⋯, 5} for the 16QAM signal with and without JCEC are shown in Figure 3. It is obvious that when JCEC is used, the corrected local cumulant estimate has a much better quality than the original without JCEC. Correspondingly, we can conclude that the main improvement is achieved for the first 6–7 sensors included in the fusion process. Furthermore, the larger gains are achieved for the lower sample sequence lengths although the better cumulant estimate quality (i.e., almost perfect for the larger number of sensors) is achieved for the higher sample sequence lengths.

Since the JCEC is based on the maximum-ratio combining using the known local SNRs, its use suppresses the influence of the sensors with the lower SNR. However, as the knowledge of the MPF channel state is assumed unknown, the discrimination between MPF channels (i.e., associated sensors) with different channel lengths is not supported [25]. Hence, it is expected that the better cumulant estimate improvements are achieved with the JCEC for the set of sensors that receive signal over the MPF channels with the smaller channel lengths [25], i.e., for local L∈{2,⋯, 5} compared to local L∈{2,⋯, 10}. Moreover, one has to notice that JCEC is applied in the FC, where all local cumulant estimates are collected, and thus cannot be used for HDF methods.

### 2.4. The non-Idealized Application Scenario for the Fusion Based Cooperative MC

The presented centralized fusion for the cooperative cumulant-based MC achieves maximum performance only if the assumptions used in the construction of the considered fusion methods are strictly met, i.e., only when the MC with the ICEM is used accompanied with the actual references estimated with the ICEM for the actual MPF channels and actual processing parameters [24,25,26]. However, the use of the ICEM is not possible in practice since the properties of the actual MPF channels (channel lengths and statistical properties, i.e., PDF of the channel coefficients), cannot be *a priori* known or reliably estimated in the non-cooperative MC environment from the relatively short signal sample. It should be noticed that even for MC with ICEM the actual references have different values for different MPF channels [27,29].

Consequently, when the real-world implementation is considered the cumulant estimate must be produced by using MC with BCEM. The given conclusion implies that a certain deterioration of the cumulant estimate quality, and as a result, a considerable decrease in the MC performance occurs due to errors introduced by BCEM. Furthermore, since the statistical properties of the actual MPF channels (i.e., actual MPF channel PDF type and parameters, MPF channel length L) are simply unknown in advance, we cannot rely on using actual references (the actual cumulant means and variances, actual CMs) properly estimated in accordance to the actual MPF channel (i.e., the channel that occurs for the given sensor at a given time). Therefore, we are constrained to use a mismatched set of references with the penalty of the further decrease of cooperative MC performance [24,25,26].

In fact, in order to design a universal solution, one should observe the worst case scenario of mismatched references. Thus, we have to use the theoretic means (see Table 1) as the reference cumulant means. The reference CMs and reference cumulant variances are acquired by averaging (over different L) the actual CMs and the actual cumulant variances estimated for the theoretic means and by using the MC with ICEM. The averaging over different L is performed in order to obtain independence regarding the unknown MPF channel properties. Finally, the cumulant estimate for any given receiver is made by using the MC with BCEM. The previously described case is here considered as the non-idealized application scenario (NIAS). The NIAS is also considered in Section 4 for the estimation of cooperative MC performance (with the centralized or the distributed fusion) for all considered MC solutions.

## 3. A Distributed Hybrid two-Stage Fusion for Cooperative MC

Recent studies [20,21,22,23,24,25,26,27,28,29] have shown that the previously described centralized fusion for cooperative modulation classification (MC) facilitates considerable performance gains in comparison to the classic MC solutions with a single sensor deployed. This performance enhancement is based on the fact that centralized fusion exploits the complete information about the unknown signal gathered through uncorrelated reception over the independent MPF channels. Thus, the joint decision fusion (JDF) and soft decision vector decision fusion (SDVDF) methods that use local MC features (i.e., cumulants) in the fusion process outperform the hard decision fusion (HDF) methods in which a partial loss of information occurs when the local decisions are made separately at individual sensors [24,25,26]. However, the main MC performance loss in the practical non-idealized application scenarios (NIAS) for all cooperative solutions is primarily caused by the unreliability of references used in the fusion process, i.e., the mismatch between practically available NIAS references and the optimal actual references that could be used if the actual MPF channel properties (the statistical properties and length L) were exactly known at each sensor. In essence, the use of mismatched references cause the additional loss of information that differently affects fusion methods. The JDF and SDVDF methods are less sensitive to the reference mismatch than HDF methods and thus present considerably better solution in practical applications [24,25,26]. Moreover, as this reference mismatch can be alleviated through the improvement of the cumulant estimate quality, deployment of the longer symbol sequences ($N_{S}$) and the joint cumulant estimate correction (JCEC) offer some MC performance improvement at the cost of increase in complexity. It is found that this improvement is significant only for the low quality of local cumulant estimates (i.e., low SNR, short symbol sequences, large multipath fading channel lengths) and limited (saturated) when the number of sensors is increased [25,26]. Finally, the previous studies suggest that the major MC performance gains of the cumulant-based cooperative MC solutions are achieved with the relatively small number of sensors (three to seven sensors) [25,26], especially in the previously defined scenarios with the low cumulant estimate quality.

**Figure 4:**
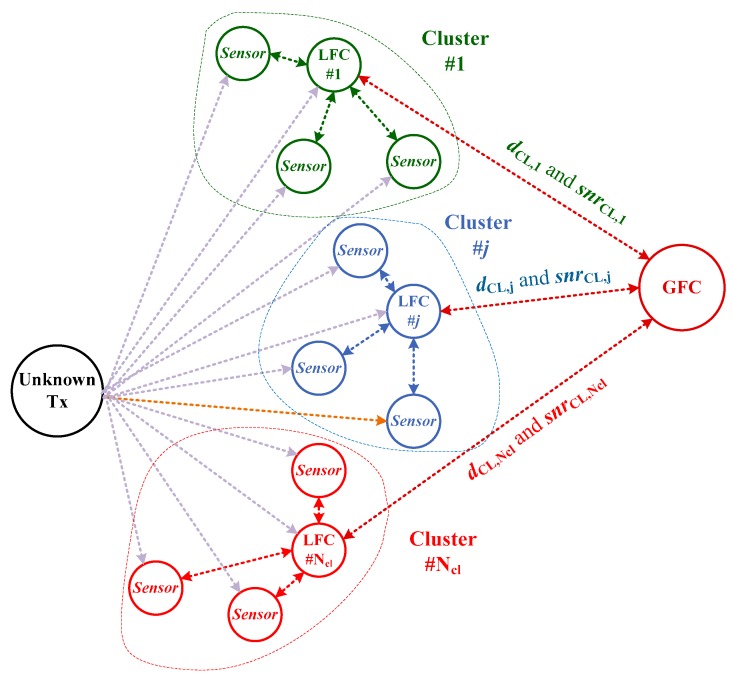
The general scheme of the cooperative MC with distributed two-stage fusion.

Considering a preceding discussion, we here propose the distributed hybrid two-stage fusion (DHyTSF) for cooperative MC (presented in Figure 4) with the aim to further mitigate the problem of mismatch references that cause the main loss in cooperative MC performance. In the DHyTSF scheme neighbouring sensors (i.e., sensors that detected and received the observed signal) form clusters, with $N_{CL}$ clusters, $N_{sen,j},j=1,\cdots ,N_{CL}$, sensors in each of the cluster, and $N_{sen}=\sum_{j=1}^{N_{CL}}N_{sen,j}$ sensors employed in the whole network. In each cluster, one of sensors acts as the local fusion center (LFC), using the same fusion method to get the cluster decisions, $d_{CL,j},j=1,\cdots ,N_{CL}$, and to compute the average SNR in cluster, $snr_{CL,j},j=1,\cdots ,N_{CL}$. These local MC results are gathered at the single global fusion center (GFC) where the final decision is made through the use of the HDF method.

The main idea of the proposed hybrid fusion is to restrict the use of mismatched references to the first stage of the DHyTSF scheme, in which the neighbouring sensors are employed in clusters and the LFC use the chosen fusion method to make the independent cluster decisions. All clusters use the same fusion method, the data fusion (DaF) or the soft decision fusion (SDF) method defined for the centralized fusion, with the mismatched NIAS references. The clusters should consist of at least four sensors in order to enable good MC performance in the cluster, since such cluster size allows the minimum sufficient MC performance gains when the fusion methods and JCEC are applied [24,25,26]. In the case of high cumulant estimate quality there is no obvious upper limit on the number of sensors that can be used in the cluster (e.g., see Figure 7). For the low cumulant estimate quality, the MC performance in the cluster is saturated with six to seven sensors in cluster (e.g., see Figure 9). Thus, additional sensors in the cluster would increase complexity but are not expected to increase MC performance. In the second stage of the proposed DHyTSF scheme, the optimal HDF (OHDF) method is used at GFC to make a final decision, but with the reliable reference CMs that are previously evaluated for the considered fusion method, the number of sensors in cluster, the average SNR in cluster, and with the mismatched (NIAS) references used—i.e., under the exact application conditions that are actually met in the first stage of the DHyTSF scheme. Therefore, in the second stage we use the OHDF method with the reference CMs that are highly reliable, and thus allow very successful fusion in this second stage. Therefore, we limit the information loss that occurs due to the use of mismatched references only to the first stage of the proposed DHyTSF scheme.

We here argue that the proposed DHyTSF scheme, with the small number of sensors using JDF or SDVDF method for intra-cluster fusion with the mismatched NIAS references (first stage) and the OHDF method used for inter-cluster fusion with the reliable references (second stage), should in fact outperform the centralized fusion with the mismatched NIAS references and the same number of sensors applied. This expectancy should be realized when there is a low quality of the local cumulant estimates (e.g., low SNR, short symbol sequences, and large multipath fading channel lengths), i.e., when the centralized fusion cannot take the full advantage of the additional information acquired by using the large number of sensors due to the considerable information loss caused by the mismatched references. Actually, the DHyTSF scheme enables a trade-off between the lower number of sensors used in the first stage (i.e., less information to get the cluster decisions than in the centralized fusion) and the more successful fusion process in the second stage with the more reliable references (i.e., the controlled information loss due to mismatched references). Therefore, when the cooperative MC is considered, conversely to the usual assumption, the distributed fusion can outperform centralized fusion in some application scenarios. The considered distributed and centralized cooperative MC schemes have similar and low computational complexity. The increase in complexity for distributed fusion when compared to the centralized one is very low. However, the distributed approach demands that the fusion process is implemented in sensor nodes. However, the low complexity allows implementation of intra-cluster fusion by using the constrained devices that are found in wireless sensor networks environments.

Moreover, due to its distributed architecture some additional benefits could be enabled, such as the lower communication resources, energy, and complexity costs. These benefits would have great importance for DHyTSF application in large-scale sensor networks. However, in order to confirm these benefits, a detailed specification of data exchange and distributed computation protocol is needed, as well as the experimental comparison with the state of the art. However, in this paper we are focused on the theoretical framework rather than specific protocol details, with the main goal to properly introduce distributed cooperative MC and show that it can achieve similar or better MC performance in comparison to centralized cooperative MC under the same conditions. Thus, a detailed framework and protocol proposal is beyond the scope of this paper.

## 4. Numerical Results

Comprehensive Monte Carlo experiments have been used in order to estimate modulation classification (MC) performance of the centralized and the distributed cooperative MC schemes. The general measure of the MC performance used was the average probability of the correct classification ($P_{CC,avg}$) defined as the averaged value of correct classification over equiprobable modulated types under the given experiment conditions [3,9,10,11,12,13,14,15,16,17,18,19,20,21,22,23,24,25,26,27,28,29,30,31,32,33,34,35,36,37,38]. The numerical analysis is performed in the form of comprehensive computer-based simulations in the MATLAB programming environment. The MC performance is estimated for the cooperative MC with the centralized fusion realized by using joint decision fusion (JDF) and soft decision vector decision fusion (SDVDF) methods, with or without joint cumulant estimate correction (JCEC) as the reference methods, as well as for the distributed fusion based on the proposed DHyTSF scheme with the JDF and SDVDF methods (with or without JCEC) used in the first stage.

The mixture of modulated signals $M_{mod}=\left\{BPSK,\;QPSK,\;16QAM,\;64QAM\right\}$, are generated as the normalized unit energy zero-mean random processes with the randomly generated symbol sequences x(n), $n=1,\cdots ,N_{S}$ with $N_{S}\in \left\{500,\;1000,\;2000,4000\right\}$, and for SNR∈[0 dB,20 dB]. The multipath fading (MPF) channels are observed with channel coefficients randomly generated according to the previously defined channel model (in Section 2.1) and for the given channel lengths (see Section 4.1). The cumulant-based MC with the blind channel estimation method (BCEM) is used as defined in Section 2.1. We used the iterative estimation procedure with a basic block formed from 5000 trials defined for each input sequence length and modulation type, while a network with up to 20 sensors and randomly generated MPF channels, local signal-to-noise ratio (SNR), input sequences $x_{i}\left(n\right),$ and additive white Gaussian noise (AWGN) is considered. These basic blocks are processed under the assumption of the non-idealized application scenario (NIAS) references for all the considered fusion methods (i.e., in the centralized fusion and in the first stage of the distributed fusion), and the properly estimated reference confusion matrices (CM) (see Section 4.2) used in the second stage of the distributed fusion. The $P_{CC,avg}$ has been evaluated for each basic block, scenario, and centralized/distributed cooperative MC scheme (for different fusion method), with the iterative procedure stopped when the maximum absolute differences for all aggregated $P_{CC,avg}$ curve lower than $5\times 10^{-3}$ for the successive basic blocks was detected.

### 4.1. Considered Sensor Network Scenarios

We here observed the centralized and distributed cooperative MC for tree sensor network scenarios defined for different distributions of sensor locations (around the unknown transmitter) in a given geographical area and the parameters of associated MPF channels. Considering the non-cooperative nature of MC, the local SNR at each sensor depends on the distance to the unknown transmitter and corresponding antenna gains (at transmitter and sensor), while the MPF channels may have different channel lengths for different sensors and are assumed mutually independent (i.e., the mutual distance between sensors is large enough to ensure uncorrelated radio signal reception).

We observe large-scale network with large number of sensors placed (distributed) over the wide geographical area. The location of the unknown transmitter has to be assumed as random in the given area, since we want to support detection and classification of any active transmitter. If we define the minimum SNR value (that depends on sensor distance to the transmitter) that is acceptable for MC purposes, we in fact define an area around the transmitter in which the sensors that receive signal with acceptable SNR are located. If this minimum acceptable SNR increases the given area (and thus number of sensors in the area) decrease in size, and vice versa. Therefore, this minimum acceptable SNR must be chosen as large enough to allow us to have the wanted number of sensors chosen for cooperative MC. If we analyze Figure 2, where the actual cumulant estimate means are given as a function of SNR, MPF channel length and symbol sequence length, we can conclude that the lowest SNR value that is suitable for MC purposes is zero dB. Below this value, the cumulant estimate quality is extremely low for all MPF channel lengths and symbol sequence length. In addition, we can see saturation in all given curves for 20 dB, which means that cumulant estimate quality, and thus MC performance, are not significantly improved for SNR values above 20 dB. Moreover, we can notice that for SNR values lower than 5 dB we have fast decrease in cumulant estimate quality, while for SNR values larger than 15 dB saturation in cumulant estimate quality occurs.

The above conclusion is used when we defined SNR interval [0 dB,20 dB], as an interval that allows us to observe change in performance for different scenarios. Of course, if we shift this interval towards the greater value, we can expect better MC performance (due to the increased cumulant estimate quality), but with the smaller area in which deployed sensors can be located. This could be appropriate if we have large number of sensors with dense distribution in the given area. Of course, if the SNR interval is shifted towards the lower values we can expect the opposite.

However, we here observed network scenarios in which the sensors are grouped in clusters, formed by the neighbouring sensors (the group of sensors that are relatively close), which are generally spaced in different directions and distances around the unknown transmitter. Therefore, we assumed that all sensors in each cluster receive the signal with the similar local SNR values in the *j*-th cluster $SNR_{j,i},\;i=1,\cdots ,N_{sen,j}$, uniformly distributed around the mean value in cluster $SNR_{j},\;j=1,\cdots ,N_{CL}$, i.e., $SNR_{i,j}\in \left[SNR_{j}-2\;\mathrm{dB},SNR_{j}+2\;\mathrm{dB}\right]$. Similarly, the sensors in each cluster receive the signal via the MPF channels of similar lengths, with the channel lengths in *j*-th cluster $L_{j,i},\;i=1,\cdots ,N_{sen,j}$, uniformly distributed around the mean value in cluster $L_{j},j=1,\cdots ,N_{CL}$, i.e., $L_{i,j}\in \left[L_{j}-1,L_{j}+1\right]$. We here considered the MPF channels with the channel lengths $L_{i,j}\in \left\{2,\cdots ,\;L_{max}\right\}$, for the two different values $L_{max}=5$ and $L_{max}=10$, in order to model dispersive MPF environments with the different delay (time) spreads, and with the mean value in cluster $L_{j},j=1,\cdots ,N_{CL}$, randomly generated for the defined $L_{max}$.

**Figure 5:**
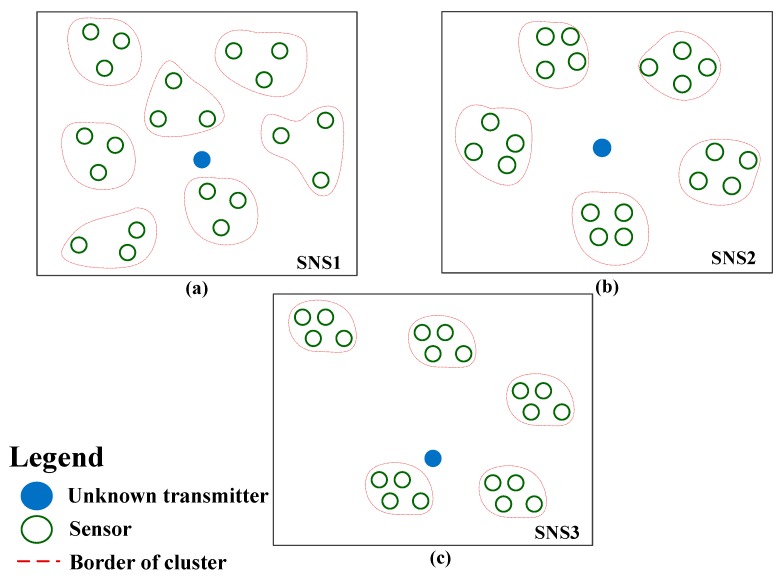
Different sensor network scenarios depending on the cluster spacing around a transmitter, (**a**) SNS1—Clusters are spaced in different directions and on different distances from the transmitter, (**b**) SNS2—Clusters are spaced in different directions but have similar distance from the transmitter, (**c**) SNS3—Clusters have different decreasing distances from the transmitter.

In order to model different possible scenarios depending on the cluster spacing around the transmitter, we used three sensor network scenarios, depicted in Figure 5, and defined as follows:

In the first sensor network scenario (SNS1), the clusters are spaced in different random directions and on different random distances around the transmitter, but with similar propagation conditions for all sensors in each cluster. This scenario is modelled with the mean SNR value of cluster $SNR_{j},\;j=1,\cdots ,N_{CL}\;$, generated as mutually independent random variables over all clusters such that $SNR_{j}\in \left[2\;\mathrm{dB},18\;\mathrm{dB}\right]$;

In the second sensor network scenario (SNS2), all clusters are assumed to have similar distance from the transmitter and thus exhibit the same mean SNR value in cluster $SNR_{j}=5\;\mathrm{dB}$ or $SNR_{j}=15\;\mathrm{dB}$, $j=1,\cdots ,N_{CL}$. These two values are chosen in order to model the reception with low and high SNR for all sensors. We here adopted SNR values after which the behaviour of cumulant estimate quality significantly change (as discussed);

The third sensor network scenario (SNS3), is a specific case in which the interval [0 dB,20 dB] is divided into $N_{CL}$ non-overlapping subintervals, with the mean SNR value in cluster $SNR_{j},j=1,\cdots ,N_{CL}\;$, defined as a mean subinterval value, starting from the lowest value for j=1. The scenario SNS3 models the case in which the cluster distances from the transmitter decrease for every next cluster added (and thus local SNRs increase), i.e., the case of uniformly distributed cluster distance from the transmitter.

According to the defined sensor network scenarios, in each trial of performed Monte Carlo experiments we generated average cluster values (the mean SNR value in cluster $SNR_{j},$ and the mean MPF channel length in cluster $L_{j},\;j=1,\cdots ,N_{CL}$), as random or static value (depending on the sensor network scenario). After that, local MPF channel lengths ($L_{i,j}$) and local SNR values ($SNR_{i,j}$) for each sensor are generated as uniformly distributed discrete random variables in interval defined by average cluster values. The distance between sensors is assumed to be large enough to ensure reception over uncorrelated MPF channels, and thus the MPF channel coefficients for each sensor are generated as mutually independent according to the model for dispersive MPF channel defined at the end of Section 2.1.

### 4.2. The Estimation of Reference CMs Used in the Second Stage of the DHyTSF Scheme

In order to estimate reference CMs used in the second stage of the distributed hybrid two-stage fusion (DHyTSF) scheme we estimated the performance of the cooperative MC with the centralized fusion for JDF and SDVDF methods (with and without JCEC) for the network of two to 10 sensors. We used the procedure described in the first paragraph of Section 4 for the SNS2 scenario but for the complete set of mean SNR values, i.e., $SNR_{j}\in \left[2\;\mathrm{dB},18\;\mathrm{dB}\right]$. In order to make the estimated CMs more robust we averaged the CMs for all mean cluster channel lengths, $L\in \left[3,L_{max}-1\right]$ for $L_{max}=5$ or $L_{max}=10$. Moreover, in order to generate CMs references that are not adjusted for the MPF channel model that we used in the numerical analysis, we repeated this procedure by changing the variance $\sigma_{h,i}^{2}$ of the defined MPF channel model (see Section 2.1) over the set 0.01, 0.03. 0.05, and 0.07 and averaged the resulting CMs. This way, due to the averaging of the CMs references for the different parameters of the MPF channel model (i.e., different channels), we introduced the reference mismatch for the second stage of the DHyTSF scheme in order to model the more realistic application conditions.

### 4.3. Estimated Cooperative MC Performance for the Different Sensor Network Scenarios

The performed comprehensive numerical analysis produced a large amount of data for the different sensor network scenarios, MPF channel, and processing parameters, considered fusion methods with/without JCEC, etc. Yet, due to a limited space we here present only the most important and illustrative results and corresponding conclusions. To achieve a clear presentation, only the MC performance of the relevant (i.e., the most successful) fusion methods are included in figures.

**Figure 6:**
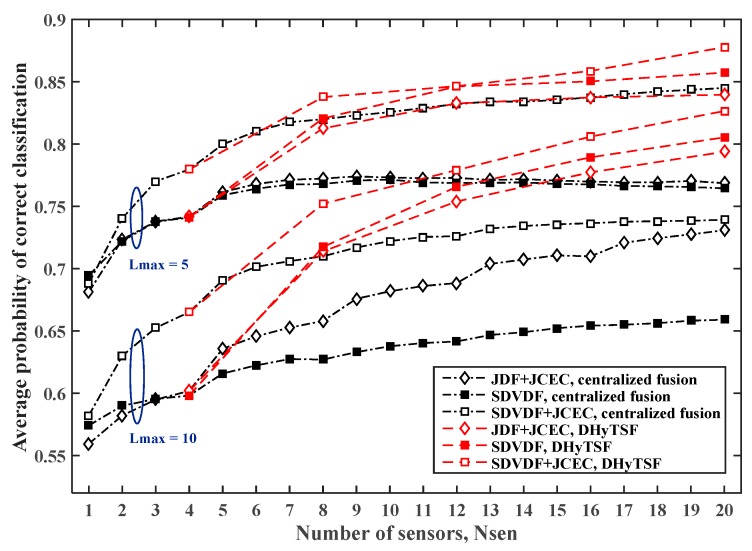
The estimated $P_{CC,avg}$ value for the centralized and distributed cooperative MC in SNS1, for $N_{S}=500$ and different dispersive environments ($L_{max}=5$ and $L_{max}=10$ ).

**Figure 7:**
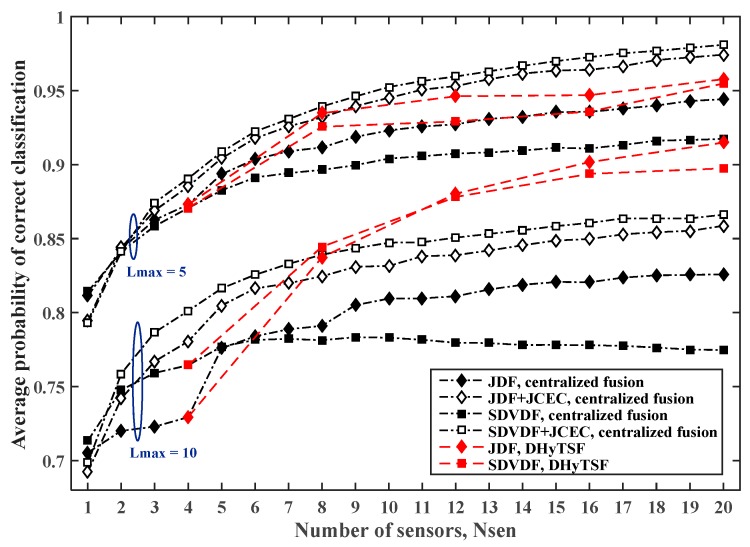
The estimated $P_{CC,avg}$ value for the centralized and distributed cooperative MC in SNS1, for $N_{S}=2000$ and different dispersive environments ($L_{max}=5$ and $L_{max}=10$ ).

The estimated $P_{CC,avg}$ for the centralized and distributed cooperative MC in SNS1 with the random average SNR cluster values (i.e., distance from transmitter) and $N_{CL}=5$ clusters of equal size (four sensors by cluster), the symbol sequence length $N_{S}=500$ and $N_{S}=2000$ are given in Figure 6 and Figure 7, respectively, for the different dispersive environments (i.e., $L_{max}=5$, $L_{max}=10$).

In the case of the low cumulant estimate quality, i.e., short sample sequence ($N_{S}=500$) or more dispersive MPF channels ($L_{max}=10$), due to the large mismatch of the NIAS references, the here proposed DHyTSF scheme considerably outperform centralized fusion with JCEC (4% to 10% of the absolute $P_{CC,avg}$ value). The centralized fusion without JCEC is clearly outperformed by the distributed fusion (8% to 18% of absolute $P_{CC,avg}$ value) in all observed scenarios. On the other hand, in the case of solid cumulant estimate quality, i.e., long sample sequence ($N_{S}=2000$) and less dispersive MPF channels ($L_{max}=5$), the NIAS references are much more appropriate, and hence the centralized fusion achieves better performance and outperform the distributed fusion when the large number of sensors is used. In fact, in that case the loss of information due to the use of mismatched references is not so significant and the centralized fusion enables more efficient use of the extra information gathered by using multiple sensors.

**Figure 8:**
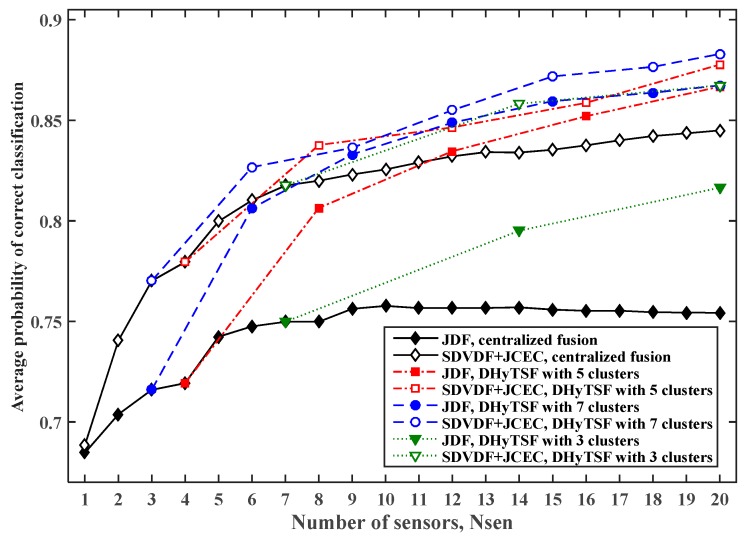
The estimated $P_{CC,avg}$ value for the centralized and distributed cooperative MC in SNS1, for different number of cluster $N_{CL}\in \left\{3,5,7\right\}$, when $N_{S}=500$ and $L_{max}=5$.

In order to estimate the influence that the number of clusters (and cluster size) has on the DHyTSF scheme performance, an additional analysis has been done. Networks with up to 20 sensors grouped in three clusters with seven sensors (the last one has six sensors), five clusters with four sensors, and seven clusters with three sensors (the last one has two sensors) has been observed. The results showed that with the increased number of clusters (a smaller cluster size) a slow improvement of cooperative MC performance is achieved but at the expense of increased communication burden and complexity. The estimated $P_{CC,avg}$ for the cooperative MC for SNS1 with different number of clusters $N_{CL}\in \left\{3,5,7\right\}$, the symbol sequence length $N_{S}=500$ and $L_{max}=5$ are given in Figure 8, as the typical case.

As seen in Figure 8, the deployment of seven clusters offers only slightly better performance than the one with five clusters, while for the smaller number of clusters, i.e., $N_{CL}=3$, a decrease in performance can be observed. This is expected, since the second stage in the DHyTSF scheme operates on reliable references and thus more successfully exploit the available information. However, when the cumulant estimate quality decreases the use of more clusters with lower number of sensors in each cluster cannot achieve reliable decisions in the first step (due to the small number of sensors). Thus, the reliability of the CMs used in the second stage rapidly decreases, which results in poor MC performance. Therefore, for the network of 20 sensor nodes, as considered in this numerical analysis, the deployment of five clusters presents an adequate choice for a broad set of application scenarios. Hence, in the further discussion in this paper only the results for a cluster with four sensors (i.e., five clusters for the network with 20 sensors) are presented. It should be noted that this is not a general conclusion. In fact, for the network with more sensor nodes clusters with more than four nodes could be employed. However, the general conclusion is that the DHyTSF scheme with the clusters that have less than four sensor nodes do not enable successful application. In that case, the low number of sensor nodes in the first stage (i.e., intra-sensor fusion) prevents the effective data fusion thus producing unreliable local cumulant decisions for the second stage.

**Figure 9:**
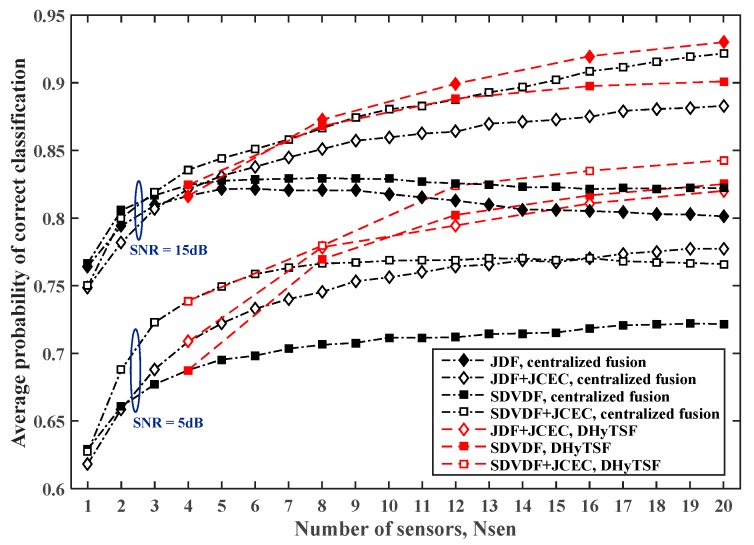
The estimated $P_{CC,avg}$ value for the centralized and distributed cooperative MC in SNS2, for $N_{S}=500$ and $L_{max}=5$.

**Figure 10:**
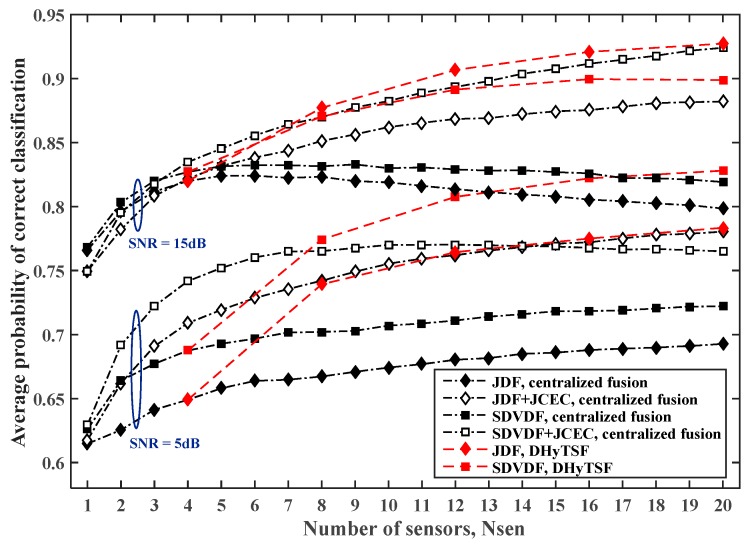
The estimated $P_{CC,avg}$ value for the centralized and distributed cooperative MC in SNS2, for $N_{S}=500$ and $L_{max}=10$.

The estimated $P_{CC,avg}$ for cooperative MC in SNS2 for network with $N_{CL}=5$. clusters of equal size when the mean SNR value in all clusters is 5 dB and 15 dB, and the symbol sequence length is $N_{S}=500$ are presented in Figure 9 and Figure 10, for the different dispersive environments defined with $L_{max}=5$ and $L_{max}=10$, respectively.

Since we use short symbol sequence length, and thus achieve a relatively low cumulant estimate quality, the distributed fusion considerably outperforms centralized fusion for the lower SNR (5% to 8% of the absolute $P_{CC,avg}$ value), while for the higher SNR similar performance can be noticed for the centralized fusion with JCEC (which improve cumulant estimate quality) and the DHyTSF scheme. The centralized fusion without JCEC is clearly outperformed by distributed fusion (8% to 15% of absolute $P_{CC,avg}$ value) in all observed scenarios. For the longer symbol sequence length, which is characterized with higher computational complexity, the DHyTSF scheme outperforms the centralized fusion with JCEC only for the lower SNR values while for the higher SNR values centralized fusion achieve better performance for the larger number of sensors.

**Figure 11:**
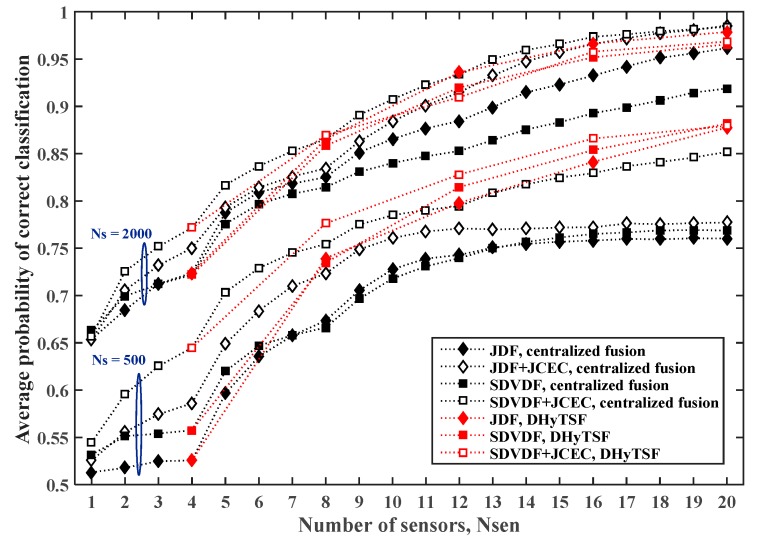
The estimated $P_{CC,avg}$ value for the centralized and distributed cooperative MC in SNS3, for $N_{S}=500$ and $N_{S}=2000$ and less dispersive environment ($L_{max}=5$ ).

**Figure 12:**
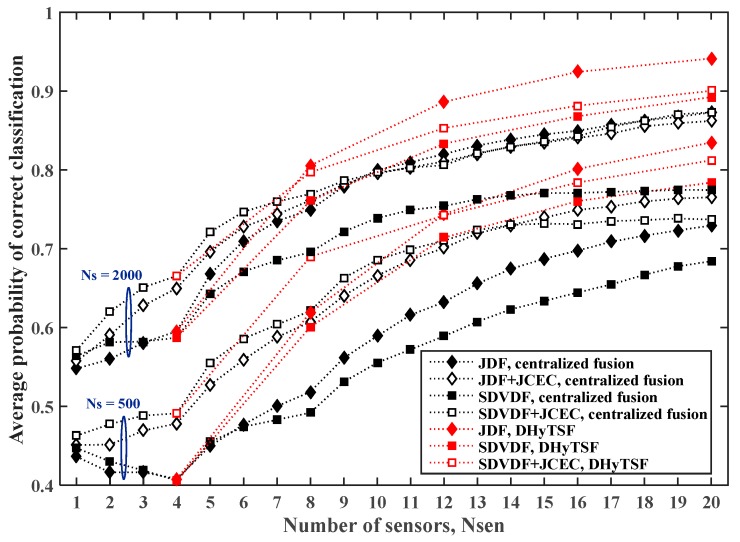
The estimated $P_{CC,avg}$ value for the centralized and distributed cooperative MC in SNS3, for $N_{S}=500$ and $N_{S}=2000$ and highly dispersive environment ($L_{max}=10$ ).

Finally, the estimated $P_{CC,avg}$ for the cooperative MC in SNS3 with $N_{CL}=5$ clusters of equal size and the symbol sequence lengths $N_{S}=500$ or $N_{S}=2000$, are presented in Figure 11 and Figure 12, for the less ($L_{max}=5$) and more ($L_{max}=10$) dispersive environments, respectively.

In the case of the less dispersive environment ($L_{max}=5$), the DHyTSF scheme slightly outperforms a centralized fusion with JCEC for the shorter symbol sequences ($N_{S}=500$), and achieves a similar performance for the longer sequences ($N_{S}=2000$). On the other hand, for the highly dispersive environment ($L_{\max}=10$), the DHyTSF scheme clearly outperforms the centralized fusion with JCEC in all scenarios (6% to 10% of absolute $P_{CC,avg}$ value). Yet again, the centralized fusion without JCEC is always outperformed by the DHyTSF scheme.

### 4.4. Overview and Discussion

The results of the numerical analysis (simulations) for all the considered scenarios are in a complete accordance with the theoretic discussion given in the previous sections. The main conclusions based on the presented data are:

- The centralized fusion achieves better performance when the JCEC is used to improve the cumulant estimate quality, and thus reduce the information loss due to the mismatched references, with the SDVDF+JCEC method being the best solution in almost all the observed scenarios;
- For the lower cumulant estimate quality (i.e., low SNR and/or short symbol sequence and/or highly dispersive MPF environment) the DHyTSF scheme (with JDF or SDVDF+JCEC in the first stage) preserves more information in the fusion process than the centralized fusion, and consequently considerably outperforms the centralized fusion;
- For the higher cumulant estimate quality (i.e., high SNR and/or long symbol sequence and/or less dispersive MPF environment), the DHyTSF outperforms the centralized fusion without JCEC (without the cumulant estimate correction) but the centralized fusion with JCEC becomes a slightly better solution as the information loss due to the mismatched references is not so high. However, even in this situation the centralized and the distributed fusion achieve similar MC performance;
- For scenarios with sensors (clusters) located at different distances from the unknown transmitter, which is the expected situation in realistic applications for large-scale networks, the DHyTSF scheme achieves larger gains compared to the centralized fusion for the lower cumulant estimate quality, and is slightly outperformed for the higher cumulant estimate quality;
- In the case of lower MC classifier complexity, i.e., shorter received symbol sequences, the DHyTSF scheme clearly outperforms the centralized fusion in the all considered scenarios.

## 5. Conclusions

The cooperative modulation classification (MC) with the centralized fusion is reported to offer significant performance gains in comparison to single sensor classifiers [22,23,24,25,26,27,28,29,30,31,32,33,34,35,36,37,38], especially for the dispersive MPF environments. Due to the inherent spatially distributed sensors/radios wireless sensor networks (WSN) and cognitive radio networks (CRN) represent the natural environments for cooperative MC. CRN presents a new application field for modulation classification, while cooperative MC is mainly observed as an additional feature in spectrum sensing that can facilitate improvement of overall CRN performance [3,6,20,31,37,38]. On the other hand, WSN presents supporting network technology that enables the design of specific WSN for applications such as cooperative sensing, detection, identification, MC, and localization, realized by employing standard communication interface for the wireless networking and additional radio receiver used as sensor [21,22,23,24,25,26,27,28,29,30].

So far, two approaches to centralized cooperative MC are proposed. The first approach is feature based (FB) cooperative MC [19,22,23,24,25,26,28,29,30,36,37,38], with the cumulant-based classifiers usually considered as the convenient, simple, and robust solution for dispersive multipath fading (MPF) environments. The second approach is likelihood-based (LB) cooperative MC [21,23,27,31,32,33,34,35], with signal fusion based solutions [31,32,33,34,35] reported as able to achieve nearly optimal performance under ideal conditions and outperform the cumulant-based solutions but with the significantly increased computational complexity. However, centralized LB cooperative MC with signal fusion, require reception of the same symbol sequence by all sensors, and delivery of these sequences to the fusion center [23,31,32,33,34,35]. This results with the significant communication load between the sensors and fusion center, and demand a certain level of sensing synchronization on the network level (which also increase complexity). Thus, these solutions are quite appropriate when the sensing network is in the vicinity of the fusion center. However, significant performance loss in the case of unideal application conditions (i.e., low quality estimation of parameters assumed known) is reported [31,32,33,34]. In the case of large-scale ad hoc wireless networks, with the large number of sensors deployed in the wide area, centralized signal fusion demand high communication capacity across the network (to deliver signal samples from sensors to fusion center). Moreover, synchronized sensing of the unknown signal by using widely dispersed sensors becomes more complex as the network size increases. On the other hand, suboptimal cumulant-based cooperative MC, does not require synchronized sensing and demand only small amounts of data to be delivered to the fusion center (i.e., local MC results). Thus, we have obvious trade-off between signal fusion and cumulant-based centralized cooperative MC in terms of MC performance, communication burden, and complexity.

However, previous studies [24,25,26] showed that large MC performance loss for the centralized fusion exists due to the unreliable (mismatched) references in realistic application conditions. In order to overcome this problem, we designed a novel distributed hybrid two-stage fusion (DHyTSF) cooperative scheme. The proposed distributed cooperative MC was designed as modification of cumulant-based centralized cooperative MC in [25]. Presented numerical results for the proposed DHyTSF scheme, acquired through extensive Monte Carlo experiments, confirm the main assumptions and expectations given in Section 3. Thus, for intended application scenarios defined by low cumulant estimate quality, the DHyTSF scheme outperforms corresponding centralized scheme. Consequently, the proposed DHyTSF scheme is shown to facilitate preservation of information during the fusion process and thus achieve considerable MC performance gains over the centralized fusion for the application conditions with low cumulant estimate quality, especially when the sensors are spaced at different distances from the observed transmitter. Furthermore, the proposed DHyTSF scheme obtains similar performance as centralized MC for application conditions with high cumulant estimate quality. However, the additional analysis is needed to define the optimum cluster size and number for the network with the higher number of nodes than the one considered here.

Furthermore, the proposed DHyTSF scheme can present a more suitable solution than the centralized one for large-scale ad hoc wireless networks, due to the intuitive expected lower communication burden compared to the cooperative MC with the centralized fusion. On the other hand, the DHyTSF scheme demands support for cluster formation and intra-cluster communication, which is expected to increase operation complexity, while centralized fusion does not have such demands. However, a future work is needed to develop a detailed specification of the data exchange and distributed computation protocol as well as the experimental comparison in order to support the given claims. Moreover, further development and analysis should be performed to design a complete network solution able to support the here proposed DHyTSF scheme. The further study should be dedicated to a detailed framework and protocol proposal, including appropriate clustering and data exchange protocols, as well as the communication and computation complexity analysis.

It should be noticed that the proposed distributed scheme is not limited to cumulant-based cooperative MC, since the similar solution could be designed and analysed for different feature-based modulation classification methods. In that sense, any classifier that can achieve stable operation in intra-cluster fusion (i.e., elements of confusion matrices have low variance), present a good candidate for the distributed hybrid fusion. In addition, further MC performance improvement of the here proposed DHyTSF scheme could be achieved without a high increase in complexity if the mixture of different higher order cumulants is used for classification purposes in intra-cluster fusion. In that manner, the cooperative classification framework using maximum likelihood combining algorithm proposed in [37,38] could prove as a good choice.

If we compare the here proposed DHyTSF scheme with the centralized LB cooperative MC with signal fusion we have the similar trade-off as for the centralized cumulant-based cooperative MC discussed before. We can expect that centralized LB cooperative MC with signal fusion achieves the better overall MC performance, but at the cost of increased computational complexity. In the case of large-scale networks, the DHyTSF scheme demands much less data to be transmitted (since only local MC results are exchanged and not the complete signal samples over the network). Moreover, DHyTSF scheme does not demand synchronized sensing as centralized LB cooperative MC with signal fusion. However, DHyTSF scheme demands proper mechanisms for cluster generation and local intra-cluster data exchange.

Finally, the here proposed general DHyTSF architecture could be used as a basis to design a distributed LB cooperative MC with signal fusion. In such a solution, likelihood-based cooperative MC with signal fusion could be applied for intra-cluster fusion. Thus, the designed solution could have potential to achieve the good MC performance and at the same time resolve some of the issues that exist for centralized LB solution (the large amount of data would be delivered inside the cluster instead of across the network to the global fusion center).

## Figures and Tables

**Table 1:** The theoretic normalized fourth-order cumulant for some phase shift keying (PSK) and quadrature amplitude modulation (QAM) signals [14].

**Signal**	**BPSK**	**QPSK**	**16QAM**	**64QAM**
Label	$m_{1}$	$m_{2}$	$m_{3}$	$m_{4}$
$C_{42}^{m_{i}}$	−2.0000	−1.0000	−0.6800	−0.6191

## References

[1] Wymeersch H., Lien J., Win M.Z. (2009). Cooperative Localization in Wireless Networks. Proc. IEEE.

[2] Chen S., Zhang J., Mao Y., Xu C. (2019). Efficient Distributed Method for NLOS Cooperative Localization in WSNs. Sensors.

[3] MacKenzie A.B., Athanas P., Buehrer P.M., Ellingson S.W., Hsiao M., Patterson C., da Silva C.R.C.M. (2009). Cognitive radio and networking research at Virginia Tech. Proc. IEEE.

[4] Ganesan G., Li Y.G. (2007). Cooperative Spectrum Sensing in Cognitive Radio Part I: Two Users Networks. IEEE Trans. Wirel. Commun..

[5] Ganesan G., Li Y.G. (2007). Cooperative spectrum sensing in cognitive radio Part II: Multiuser Networks. IEEE Trans. Wirel. Commun..

[6] Do T.-N., An B. (2014). Cooperative Spectrum Sensing Schemes with the Interference Constraint in Cognitive Radio Networks. Sensors.

[7] Lataief K.B., Yhang W. (2009). Cooperative communications for cognitive radio networks. Proc. IEEE.

[8] Chen Z., Ma M., Liu X., Liu A. (2017). Reliability Improved Cooperative Communication over Wireless Sensor Networks. Sensors.

[9] Dobre O.A., Abdi A., Bar-Ness Y., Su W. (2007). Survey of automatic modulation classification techniques: Classical approaches and new trends. IET Commun..

[10] Zhu Y., Nandi A.K. (2015). Automatic Modulation Classification: Principles, Algorithms and Applications.

[11] Abu-Romoh M., Aboutaleb A., Rezki Z. (2018). Automatic Modulation Classification Using Moments and Likelihood Maximization. IEEE Commun. Lett..

[12] Wang F., Dobre O.A., Chan C., Zhang J. (2016). Fold-based Kolmogorov–Smirnov modulation classifier. IEEE Signal Process. Lett..

[13] Han L., Gao F., Li Z., Dobre O.A. (2017). Low complexity automatic modulation classification based on order-statistics. IEEE Trans. Wirel. Commun..

[14] Swami A., Sadler B.M. (2000). Hierarchical digital modulation classification using cumulants. IEEE Trans. Commun..

[15] Wu H.C., Saquib M., Yun Z. (2008). Novel automatic modulation classification using cumulant features for communications via multipath channel. IEEE Trans. Wirel. Commun..

[16] Orlic V.D., Dukic M.L. (2010). Multipath channel estimation algorithm for automatic modulation classification using sixth-order cumulants. Electron. Lett..

[17] Chang D., Shih P. (2015). Cumulants-based modulation classification technique in multipath fading channels. IET Commun..

[18] Zhang Y., Hua Y., Liu Y. (2017). Modulation classification in multipath fading channels using sixth-order cumulants and stacked convolutional auto-encoders. IET Commun..

[19] Forero P.A., Cano A., Giannakis G.B. Distributed Feature-Based Modulation Classification Using Wireless Sensor Networks. Proceedings of the 2008 IEEE Military Communications Conference—MILCOM 2008.

[20] Headley W.C., Reed J.D., da Silva C.R.M.C. Distributed Cyclic Spectrum Feature-Based Modulation Classification. Proceedings of the 2008 IEEE Wireless Communications and Networking Conference—WCNC 2008.

[21] Xu J.L., Su W., Zou M.C. (2010). Distributed automatic modulation classification with multiple sensors. IEEE Sens. J..

[22] Zhang Y., Ansari N., Su W. Optimal decision fusion based automatic modulation classification by using wireless sensor networks in multipath fading channel. Proceedings of the 2011 IEEE Global Telecommunications Conference—GLOBECOM 2011.

[23] Zhang Y., Ansari N., Su W. (2015). Multi—Sensor signal fusion--based modulation classification by using wireless sensor networks. Wirel. Commun. Mob. Com..

[24] Markovic G.B., Dukic M.L. (2013). Cooperative modulation classification with data fusion for multipath fading channels. Electron. Lett..

[25] Markovic G.B., Dukic M.L. (2015). Joint cumulant estimate correction and decision for cooperative modulation classification by using multiple sensors. Ann. Telecommun..

[26] Markovic G.B. Cooperative Modulation Classification by Using Multiple Sensors in Dispersive Fading Channels. Proceedings of the 22nd Telecommunication Forum 2014—TELFOR 2014.

[27] Xu J.L., Su W., Zou M.C. Asynchronous and High-Accuracy Digital Modulated Signal Detection by Sensor Networks. In Proceeding of the 2011 IEEE Military Communications Conference—MILCOM 2011.

[28] Wei S., Zhu Q. (2012). Cooperative Modulation Recognition Based on the Combination of Feature Fusion and Decision Fusion. J. Comput. Inf. Syst..

[29] Marković G.B. Centralized two-stage modulation classification by using networked sensors. Proceedings of the 24th Telecommunication Forum 2016—TELFOR 2016.

[30] Zhang Y., Ansari N., Su W. Multi-sensor signal fusion based modulation classification by using wireless sensor networks. Proceedings of the 2011 IEEE International Conference on Communications.

[31] Ozdemir O., Liu R., Varshney P.K. (2013). Hybrid Maximum Likelihood Modulation Classification Using Multiple Radios. IEEE Commun. Lett..

[32] Ozdemir O., Wimalajeewa T., Dulek B., Varshney P.K., Su B. (2015). Asynchronous Linear Modulation Classification with Multiple Sensors via Generalized EM Algorithm. IEEE Trans. Wirel. Commun..

[33] Wimalajeewa T., Jagannath J., Varshney P.K., Drozd A., Su W. Distributed asynchronous modulation classification based on hybrid maximum likelihood approach. Proceedings of the IEEE Military Communications Conference—MILCOM 2015.

[34] Zhang J., Cabric D., Wang F., Zhong Z. (2017). Cooperative Modulation Classification for Multipath Fading Channels via Expectation-Maximization. IEEE Trans. Wirel. Commun..

[35] Dulek B. (2017). Online Hybrid Likelihood Based Modulation Classification Using Multiple Sensors. IEEE Trans. Wirel. Commun..

[36] Abdelbar M., Tranter B., Bose T. Cooperative cumulants-based Modulation Classification under flat Rayleigh fading channels. Proceedings of the 2015 IEEE International Conference on Communications (ICC).

[37] Abdelbar M., Tranter B., Bose T. Cooperative Combining of Cumulants-Based Modulation Classification in CR Networks. Proceedings of the 2014 IEEE Military Communications Conference—MILCOM 2014.

[38] Abdelbar M., Tranter W.H., Bose T. (2018). Cooperative Cumulants-Based Modulation Classification in Distributed Networks. IEEE Trans. Cogn. Commun. Netw..

